# Negative Checkpoint Regulatory Molecule 2B4 (CD244) Upregulation Is Associated with Invariant Natural Killer T Cell Alterations and Human Immunodeficiency Virus Disease Progression

**DOI:** 10.3389/fimmu.2017.00338

**Published:** 2017-03-27

**Authors:** Fareed Ahmad, Esaki M. Shankar, Yean K. Yong, Hong Y. Tan, Gerrit Ahrenstorf, Roland Jacobs, Marie Larsson, Reinhold E. Schmidt, Adeeba Kamarulzaman, Abdul W. Ansari

**Affiliations:** ^1^Department of Clinical Immunology and Rheumatology, Hannover Medical School, Hannover, Germany; ^2^Centre of Excellence for Research in AIDS (CERiA), University of Malaya, Kuala Lumpur, Malaysia; ^3^Department of Medical Microbiology, University of Malaya, Kuala Lumpur, Malaysia; ^4^Division of Infection Biology, Department of Life Sciences, School of Basic & Applied Sciences, Central University of Tamil Nadu (CUTN), Thiruvarur, India; ^5^Division of Molecular Virology, Department of Clinical and Experimental Medicine, Linkoping University, Linkoping, Sweden; ^6^Department of Medicine, University of Malaya, Kuala Lumpur, Malaysia

**Keywords:** invariant natural killer T cells, 2B4, human immunodeficiency virus, inhibitory, IFN-γ, CD4

## Abstract

The CD1d-restricted invariant natural killer T (iNKT) cells are implicated in innate immune responses against human immunodeficiency virus (HIV). However, the determinants of cellular dysfunction across the iNKT cells subsets are seldom defined in HIV disease. Herein, we provide evidence for the involvement of the negative checkpoint regulator (NCR) 2B4 in iNKT cell alteration in a well-defined cohort of HIV-seropositive anti-retroviral therapy (ART) naïve, ART-treated, and elite controllers (ECs). We report on exaggerated 2B4 expression on iNKT cells of HIV-infected treatment-naïve individuals. In sharp contrast to CD4^−^iNKT cells, 2B4 expression was significantly higher on CD4^+^ iNKT cell subset. Notably, an increased level of 2B4 on iNKT cells was strongly correlated with parameters associated with HIV disease progression. Further, iNKT cells from ART-naïve individuals were defective in their ability to produce intracellular IFN-γ. Together, our results suggest that the levels of 2B4 expression and the downstream co-inhibitory signaling events may contribute to impaired iNKT cell responses.

## Introduction

Invariant natural killer T (iNKT) cells are a unique subset of T lymphocyte that bridges innate and adaptive immune responses (1). In addition to immune regulation, iNKT cell role is also implicated in several viral infections (2). In human peripheral blood, iNKT cells constitute a smaller fraction ranging between 0.01 and >1% of the total CD3^+^ T cells (3, 4). iNKT cells express invariant TCR Vα24 and Vβ11 chain, and NK cell markers such as CD161 and NKG2D (1). Unlike the classical T and B cells, iNKT cells recognize only glycolipid and phospholipid antigens of microbes presented on CD1d, a non-polymorphic major histocompatibility complex class I (MHC class I)-like molecule (5). Upon activation, iNKT cells rapidly produce large amounts of both IFN-γ (Th1) and IL-4 (Th2) cytokines to regulate host immune responses. Functionally, iNKT cells can be classified into CD4^−^ (Th1cytokine-expressing) and CD4^+^ (Th1 and Th2 cytokine-producing) subsets. The CD4^−^iNKT cells can further be divided into CD8^+^ as well as CD4^−^ CD8^−^ (double negative-DN) populations. CD8^+^iNKT cell predominantly exhibits a Th1 phenotype. Evidence suggests that CD4^+^ and CD4^−^iNKT cell homeostasis significantly impact the functional outcome of immune responses in the host (6, 7).

Negative checkpoint regulators (NCRs) such as cytolytic T lymphocyte-associated antigen-4 (CTLA-4), T-cell immunoglobulin mucin-3 (TIM-3), programmed death-1 (PD-1), 2B4 (CD244), lymphocyte activation gene-4 (LAG-3), and CD160 play a critical role in the regulation of anti-viral CD8^+^ T cell responses during human immunodeficiency virus (HIV) infection (8). Persistent antigen exposure and immune activation during chronic HIV infection lead to the upregulation of these molecules, initiating CD8^+^ T cell exhaustion and functional immune impairment (9, 10). The initiation of anti-retroviral therapy (ART) has been shown to dampen the expression of some of the regulatory molecules to normal levels (11, 12). In a latest development, NCRs such as PD-1, the T cell immune receptor with Ig and ITIM domains (TIGIT) and LAG-3 have been proposed to harness HIV persistence (13). Akin to that in CD8^+^ T cells, some of the NCRs have also been shown to suppress iNKT cell functions. For instance, upregulation of PD-1 on iNKT cells of ART-naïve individuals is associated with poor cell function (14). The NK cell receptor 2B4 belongs to the signaling lymphocyte activation molecule (SLAM) family of proteins that binds to a high-affinity ligand CD48 (15) and transduces signal via immune receptor tyrosin-based switch motif (ITSM) (16). 2B4 has previously been shown to regulate both T and NK cell anti-viral responses (17). Earlier studies have hinted that 2B4 could enhance NK and CD8^+^ T cell functions (18), and recent lines of evidence suggest that 2B4 may possess both co-stimulatory as well as co-inhibitory functions (19). For instance, a relatively low expression of 2B4 on NK cells has been shown to trigger their proliferation and eventual release of IFN-γ, while a higher expression resulted in functional suppression of HCV-specific CD8^+^ T cells (20).

Several studies have shown the depletion of iNKT cells, in particular, the CD4^+^ subset of these cells from the peripheral circulation of HIV-infected individuals (21–24). The primary cause of such depletion is reportedly attributed to the expression of HIV co-receptors, CCR5 and/or CXCR4 on iNKT cells (21, 22, 25). Of the iNKT cell subsets, the CD4^+^ appears to be highly susceptible to HIV infection (21, 23). In addition, iNKT cells have also been found to be defective in their ability to produce IFN-γ in HIV disease (14, 26, 27). Interestingly, ART follow-up studies have shown slow and poor recovery of iNKT cells although the data were insignificant during the first year of treatment (28, 29).

In contrast to the relatively commonly studied NCRs PD-1, TIM-3, and LAG-3, far less is known about the contribution of 2B4 in iNKT cell regulation in HIV disease. Here, we compared the surface levels of 2B4 expression and the associated cellular dysfunction in a well-defined cohort of HIV-seropositive ART-naïve, ART-treated, and elite controllers (ECs). We also provided evidence of elevated levels of 2B4 on the iNKT cells of ART-naïve individuals. Of the iNKT subsets, 2B4 expression was significantly higher on CD4^+^ as compared to the CD4^−^ subset. Further, iNKT cells were defective in their ability to produce IFN-γ and there was a positive correlation between 2B4^+^ iNKT cells and HIV disease progression. These data advance our understanding of iNKT cell regulation during virus insult.

## Materials and Methods

### Human Subjects

A total of 48 HIV-infected individuals including HIV-seropositive (ART-naïve, *n* = 23), combination ART-treated (cART, *n* = 19), and elite controllers (ECs, *n* = 6), and HIV-seronegative healthy controls (HCs, n = 15) were recruited into the study. Naïve individuals never received ART while treated individuals were on ART for at least one year. The ART regimens comprised three or four of the following: tenofovir, emtricitaine, nevirapine, retrovir, atazanavir, ritonavir, abacavir, zidofovir, saquinavir, fosamprenavir, lopinavir, lamivudine, efavirenz, and darunavir. A summary of clinical features of all participants is described in Table 1. Written informed consents were taken from each participant and the study was approved by the Institutional Ethics Committee (MEC) and the Institutional Review Board (IRB) of the University of Malaya Medical Center (UMMC), Kuala Lumpur, Malaysia and the Hannover Medical School, Germany, respectively.

**Table 1:** **Clinicodemographic characteristics of study participants**.

Group	Numbers (*n*)	Age-years (median)	Sex		CD4 count (SEM)	Viral load (median)	CD4/CD8 (SEM)
			M	F			
HC	15	39	10	5	NA	NA	NA
Naïve	23	38	16	7	330 ± 43.4	67,700 ± 65,935	0.370 ± 0.042
ART	19	53	12	7	528 ± 59.7	ND	0.670 ± 0.052
EC	6	54	4	2	1,066 ± 134	33 ± 59.7	0.99 ± 0.128

*CD4 count and CD4/CD8 ratio represented as mean ± SEM while viral load as median ± SEM*.

*ART, anti-retroviral treated; EC, elite controllers; F, female; HC, healthy controls; Naïve, HIV untreated; M, male; NA, not available; ND, not detected*.

### Peripheral Blood Mononuclear Cells (PBMCs) and Immunophenotyping

Peripheral blood mononuclear cells were isolated as described before (30, 31) and stored in liquid nitrogen until use. Briefly, freshly drawn intravenous blood was subjected to ficoll-hypaque gradient centrifugation. 1–2 × 10^6^ PBMCs were first stained with fixable viability dye eFluor 506 (eBiosciences) in order to exclude dead cells from the sample. APC-conjugated PBS-57-loaded CD1d tetramer (NIH Tetramer Core Facility, National Institute of Health, Atlanta, GA, USA) and a panel of following antibodies were purchased from BioLegends; PECy7-CD3, APC-Cy7-CD3, Pacific Blue-CD4, PerCP Cy5.5-CD8, FITC-2B4. Fluorescence-minus-one (FMO) stain was included to avoid false-positive signals. Cells were acquired on a BD FACSCanto II flow cytometer using FACS Diva software (v.7), and analyzed by FlowJo software (v.8.4.4, Tree Star).

### Cell Stimulation and Intracellular IFN-γ Cytokine Detection

Briefly 2–3 × 10^6^cells were stimulated with 100 ng/ml alpha-galactosylceramide (α-GalCer, kindly provided by Dr. Paul Savage, Brigham Young University, UT, USA) in a 24-well culture plate. After 1 h of stimulation, brefeldin-A (10 µg/ml) was added and cultured for another 5 h. Cells were harvested and washed twice with phosphate buffer saline (PBS) before staining with fixable viability dye eFluor 506 exclude dead cell. Cells were then surface stained with CD3, CD1d tetramer followed by fixation, and permeabilization using Fix/Perm kit (BD biosciences kit) as described before (32). Pacific Blue-IFN-γ antibody (BioLegends) was added to detect intracellular expression. Cells were washed before acquisition by flow cytometer.

### Statistical Analyses

For statistical evaluation of data, we have used GraphPad PRISM (v.5.0) software. Unpaired, two-tailed *t*-test was used to compare between two groups or one-way ANOVA followed by Tukey test was used for comparing more than two groups. To determine correlation, Pearson analysis was employed. *P* values of <0.05 were considered significant.

## Results

### CD4^+^ iNKT Cell Subset Was Preferentially Lost from the Circulation of HIV-Positive Treatment-Naïve Patients, and ART Failed to Restore CD4^+^ iNKT Cell Frequency

**Figure 1:**
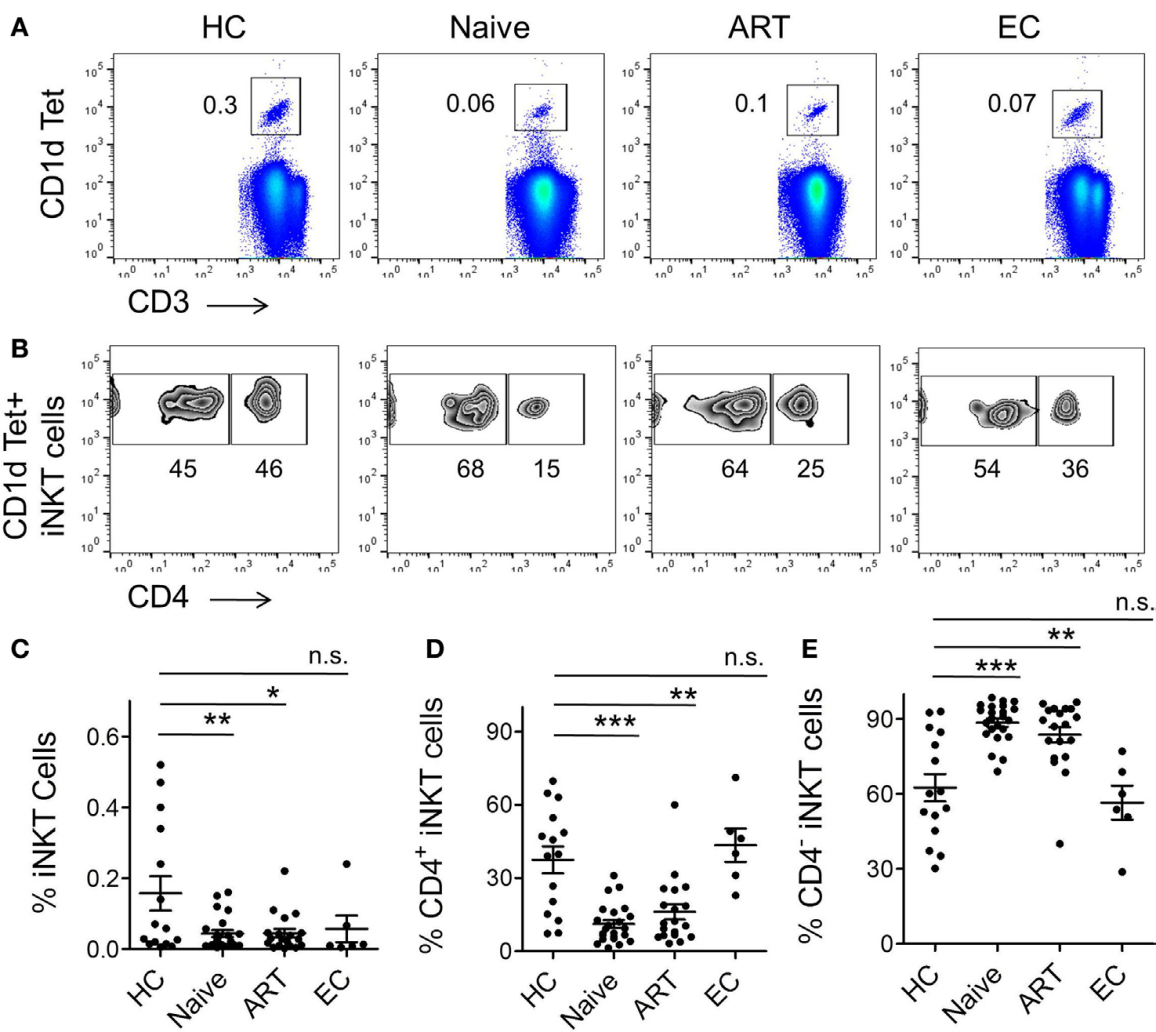
**Frequency of CD1d-restricted invariant natural killer T (iNKT) cells and their subsets**. Frozen peripheral blood mononuclear cells (PBMCs) from healthy controls (HCs, *n* = 15), human immunodeficiency virus-seropositive (ART-naïve, *n* = 23), ART-treated (ART, *n* = 19), and elite controllers (ECs, *n* = 6) were stained with PBS-57 loaded/CD1d tetramer, anti-CD3, and anti-CD4 antibodies before acquisition by flow cytometer. A detailed gating strategy is described in Figure S1 in Supplementary Material. **(A)** Representative FACS plot from an individual of each cohort, numbers indicate percent CD1d Tet^+^ iNKT cells. **(B)** Representative plot showing percent of CD4^+^ and CD4^−^ sub-population within the CD1d tet^+^ iNKT cell-gated population. **(C)** Scatter plot showing a summary of CD1d tet^+^ iNKT cells frequency across the various study cohorts. **(D)** Frequency of CD4^+^ iNKT cells and CD4^−^iNKT cells **(E)** in various cohorts. *P < 0.05, **P < 0.01, ***P < 0.001, and n.s. stands for non-significant.

To investigate the size of iNKT cell pool in the peripheral blood of different study groups, we employed a flow cytometric approach using iNKT cell-specific PBS 57-loaded/CD1d tetramer and anti-CD3 antibody (Figure 1A). Taking into consideration the scarcity of iNKT cells in peripheral blood, we applied a stringent gating strategy during cell acquisition and data analysis (Figure S1 in Supplementary Material). Fluorescence-minus-one (FMO) staining was used to determine the threshold values for expression of the specific markers. Consistent with previous reports (23, 33), a substantial loss of iNKT cells was observed in ART-naïve individuals (mean 0.04 ± 0.009%, *P* = 0.008) in comparison with healthy controls (HCs) (mean 0.16 ± 0.048%) (Figure 1C). Interestingly, the frequency in ART subjects remained low (mean 0.04 ± 0.012%, *P* = 0.017) in relation to HCs. Notably, the initiation of ART regimens was unable to completely reconstitute iNKT cell numbers. However, the frequency of iNKT cells in ECs (mean 0.06 ± 0.037%) was more or less similar as HCs (*P* = 0.502). The data from EC cohort suggest that in addition to infection of iNKT cells by HIV, on-going HIV replication may act as an additional factor contributing significantly to iNKT cell depletion from peripheral blood.

As described, iNKT cells can be broadly divided into CD4^−^ and CD4^+^ subsets (6), hence we sought to examine the frequency of these two subsets during HIV infection. We labeled the cells with CD1d tetramer, anti-CD3, and anti-CD4 antibodies and analyzed it through flow cytometry (Figure 1B). We observed severe depletion of CD4^+^ iNKT cell subset in ART-naïve subjects (mean 11.13 ± 1.65%, *P* = 0.0001) (Figure 1D). This could probably be due to HIV infection and subsequent loss of CD4^+^ iNKT cells. Interestingly, the cell frequency remained lower in the ART cohort (mean 16.11 ± 3.12%, *P* = 0.001) compared to HCs (mean 37.47 ± 5.51%), suggestive of the lack of appreciable reconstitution of CD4^+^ iNKT cells despite receiving ART. Nonetheless, the CD4^+^ iNKT cell distribution in ECs was more or less similar to that seen in HCs (*P* = 0.549).

Next, we sought to estimate the CD4^−^iNKT cell subset (Figure 1B). Interestingly, there was a marked increase in the cell frequency among ART-naïve (mean 88.60 ± 1.66%, *P* = 0.0001) individuals as compared to HCs (mean 62.51 ± 5.49%) (Figure 1E). However, we did not observe any significant impact in the ART patient group as frequency of CD4^−^iNKT cell continued to remain higher. Appearance of relatively higher CD4^−^iNKT cells is believed to be mainly contributed by CD8^+^ iNKT cells (33).

### Expression of 2B4 Was Significantly Upregulated on Bulk of CD3^+^ T Cells of ART-Naïve Individuals

**Figure 2:**
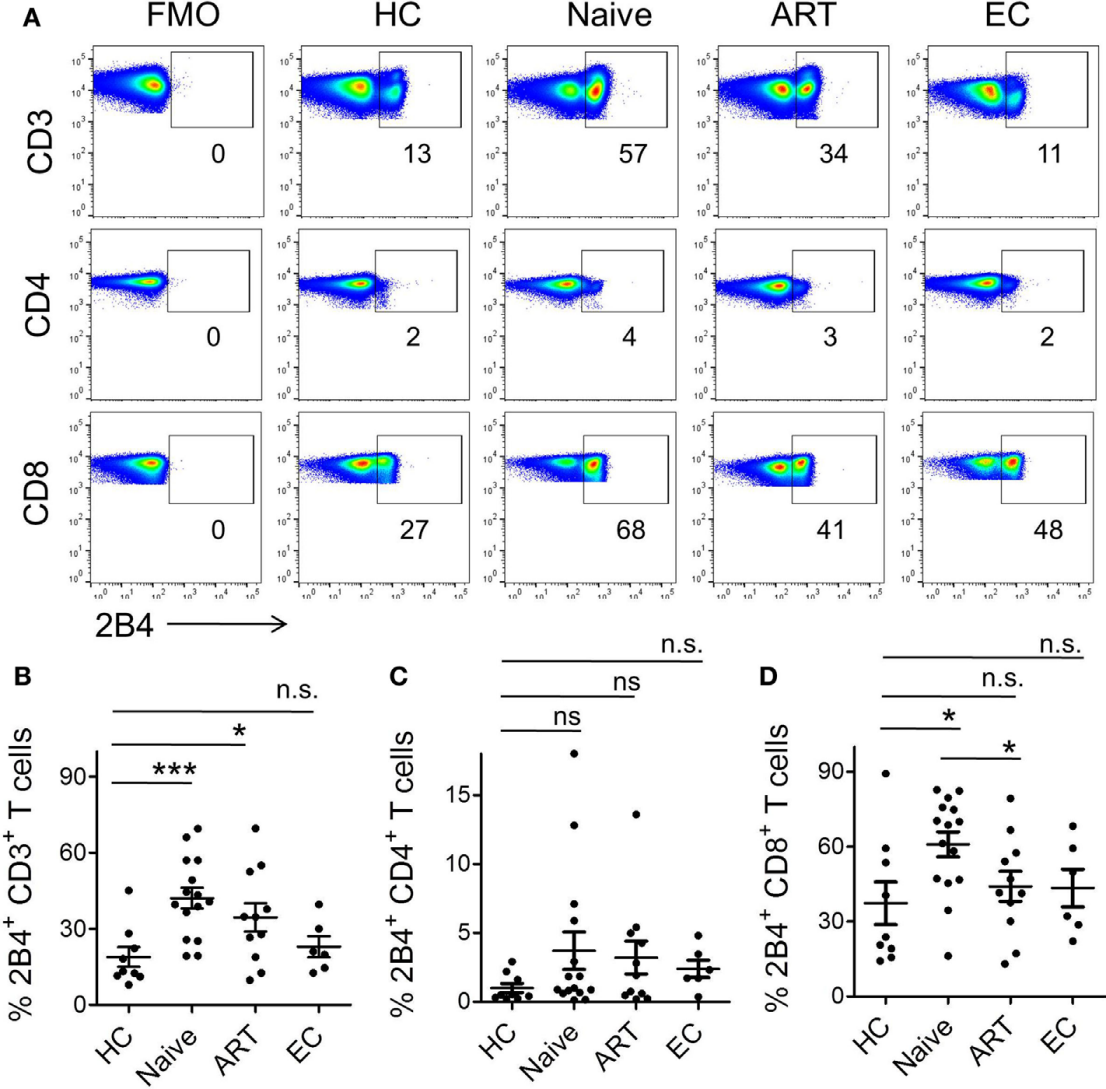
**Expression pattern of 2B4 molecule on CD3, CD4, and CD8 T cells**. **(A)** Representative FACS plots showing percent 2B4 expression on total CD3 (upper panel), CD4 (middle panel), and CD8 (lower panel) from different cohorts. Fluorescence-minus-one (FMO) staining was used to determine the threshold values for the expression of specific markers. **(B)** Scatter plot showing the mean percentage 2B4 expression on CD3^+^ T cells of various cohorts, HCs (*n* = 9), ART naïve (*n* = 15), ART (*n* = 11), and ECs (*n* = 6); whereas **(C,D)** represent the mean percentage of 2B4 expression levels on CD4^+^ and CD8^+^ T cells, respectively. *P < 0.05, ***P < 0.001, and n.s. stands for non-significant value.

Given that a complex network of co-stimulatory and co-inhibitory molecules regulate T cell immune responses (8), and that differential expression of some of the well characterized molecules (PD-1, CD160, TIM-3, LAG-3, and 2B4) has already been shown to severely affect antigen-specific CD8^+^ T-cell responses, especially in HIV and HCV (10, 34–36) infections, their expression on iNKT cells of chronic viral diseases are yet to be investigated. Given the suppressive role of 2B4 in HIV-specific CD8^+^ T cells (10), we examined the levels of 2B4 expression on T cell subsets including CD3^+^, CD4^+^, and CD8^+^ T cells (Figure 2A). The expression of 2B4 was markedly increased on bulk CD3^+^ T cells of ART-naïve (mean 42.23 ± 4.06%, *P* = 0.0009), and relatively less significantly in ART-treated individuals (mean 34.56 ± 5.58%, *P* = 0.041) as compared to HCs (mean 18.91 ± 3.89%) (Figure 2B). However, the differences of 2B4 were not significant between ART-treated and treatment-naïve individuals (*P* = 0.27), whereas ECs showed comparable expression pattern with respect to HCs.

Next, we sought to examine the level of 2B4 expression on CD4^+^ T cells. We observed very low 2B4 expression as compared to bulk CD3^+^ and CD8^+^ T cells. Further, there was no significant difference when both ART-naïve and ART subjects were compared with HCs, despite an apparent increase in 2B4 expression across both the study groups (Figure 2C). These data strongly suggest an induction of 2B4 on T cells during HIV infection. In contrast to CD4^+^ T cells, CD8^+^ T cells displayed significantly higher levels of 2B4 in ART naïve (mean 60.95 ± 5.11%, *P* = 0.01) as compared to HCs (mean 37.34 ± 8.55%) (Figure 2D). Furthermore, individuals who received ART exhibited significantly lower 2B4 (mean 44.13 ± 6.02%, *P* = 0.041) levels as compared to ART-naïve subjects. Our observations are in line with previous findings where 2B4 expressions on virus-specific CD8^+^ T cells declined to normal levels following the initiation of ART (10, 20). In general, the basal levels of 2B4 expression on CD4^+^ cells appeared to be lower than CD8^+^ T cells (Figure S2A in Supplementary Material).

### 2B4 Expression Was Higher in Treatment-Naïve Individuals and ART Initiation Failed to Normalize 2B4 Levels on iNKT Cell Subsets

**Figure 3:**
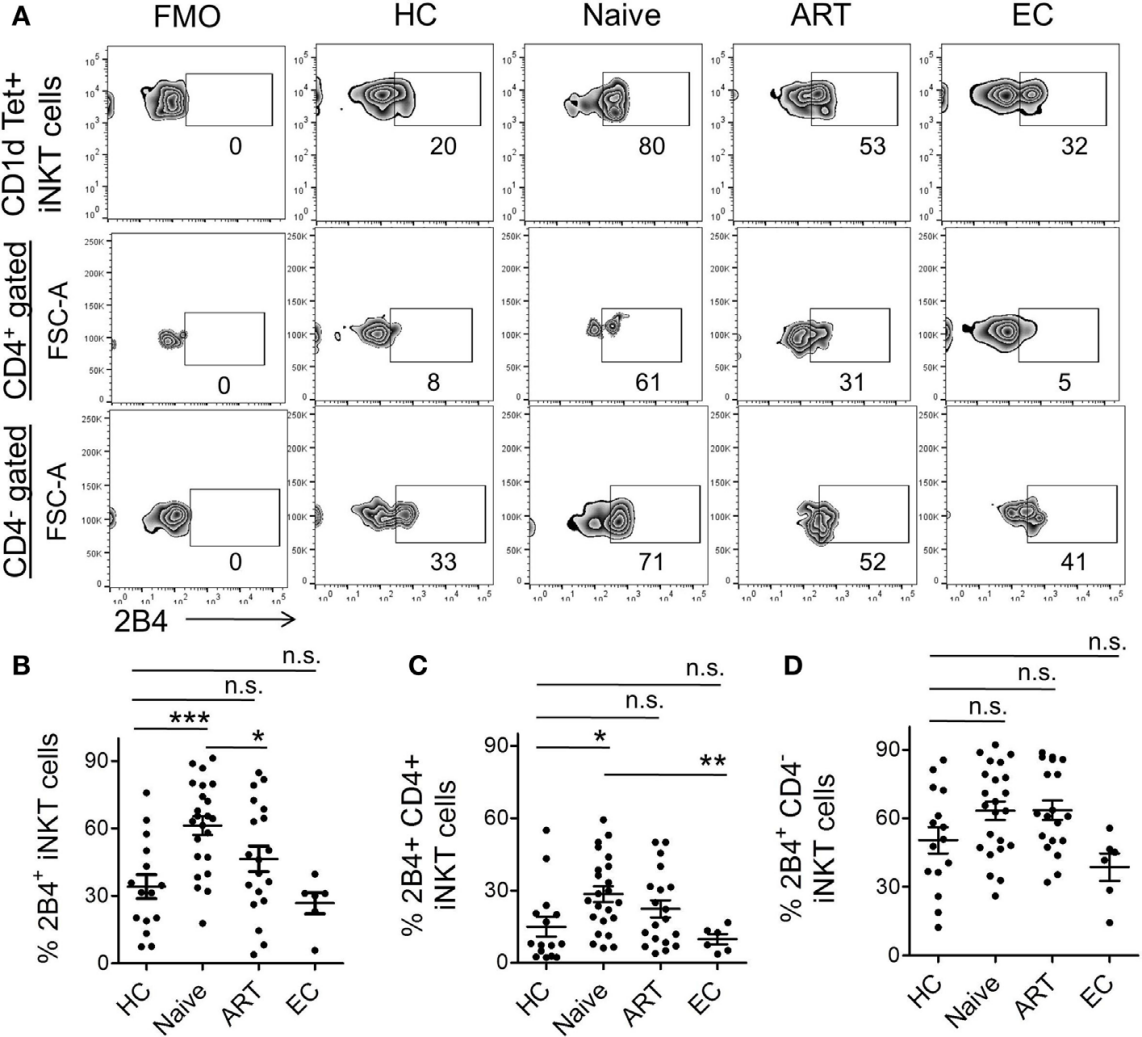
**Expression levels of 2B4 on invariant natural killer T (iNKT) cells and their subsets**. **(A)** Representative zebra plot showing percent 2B4 expression on iNKT cells of representative individuals from various cohorts. iNKT cells (PBS-57 loaded/CD1d-tetramer^+^ CD3^+^) gated population was looked for surface 2B4 levels (upper panel). While 2B4 expression on CD4^+^ (middle panel) and CD4^−^iNKT cell subset (lower panel) were first gated on CD4^+^ and CD4^−^ population then presented with FSC-A (*Y*-axis). **(B)** Scatter plot showing the mean percentage of 2B4 levels on bulk iNKT cells of HCs (*n* = 15), ART naïve (*n* = 23), ART (*n* = 19), and ECs (*n* = 6). Mean percentage of 2B4 levels on CD4^+^ **(C)** and CD4^−^iNKT cell subsets **(D)**. Fluorescence-minus-one (FMO) was taken to exclude false-positive values for 2B4. *P < 0.05, ***P < 0.001, and n.s. stands for non-significant value.

Experimental evidence suggests that 2B4 levels were elevated on CD4, CD8, and NK cells during chronic HCV and HIV (10, 20) infections. Nonetheless, the expression of 2B4 on iNKT cells has seldom been investigated. Here, we endeavored to investigate 2B4 expression using flow cytometry across the study groups. We found significantly higher levels of 2B4 expression in ART-naïve individuals as compared to HCs, ART and ECs (Figure 3A). The expression was ~2-fold higher in ART-naïve (mean 61.30 ± 4.13%, *P* = 0.0003) than HCs (mean 34.10 ± 5.34%) (Figure 3B). The ART-treated subjects showed significantly lower 2B4 levels (mean 46.47 ± 5.70%, *P* = 0.037) as compared to ART naïve indicating that treatment initiation led to marked downregulation of 2B4 expression. On the other hand, ECs with apparently suppressed HIV replication, exhibited more or less similar levels of 2B4 as compared to HCs.

Next, we determined to examine the expression of 2B4 on the different iNKT cell subsets (Figure 3A). CD4^+^ cells of ART-naïve individuals showed significantly higher 2B4 expression (mean 28.48 ± 3.24%, *P* = 0.013) relative to HCs (mean 14.94.34 ± 4.08%) (Figure 3C). Of note, ECs exhibited significantly lower levels of 2B4 as compared to ART-naïve (*P* = 0.007) subjects suggesting that lack of HIV replication failed to induce 2B4 expression on CD4^+^ iNKT cell subsets from ECs. However, we did not observe any apparent decrease in 2B4 in ART-treated individuals, suggesting that treatment initiation seldom had any effect on 2B4 expression. Although there was an increase in 2B4 in ART-naïve and ART-treated individuals as compared to HCs, this, however, failed to reach a significant value (Figure 3D). It should be noted that the baseline 2B4 expression on CD4^−^iNKT cells was always greater than that of the CD4^+^ subsets (Figure S2B in Supplementary Material). Together, our data suggest that HIV infection leads to the induction of 2B4 on bulk T cells as well as on their subsets, especially the CD4^+^ iNKT cells.

### Expression of 2B4 on iNKT Cells Inversely Correlated with Intracellular Production of IFN-γ

**Figure 4:**
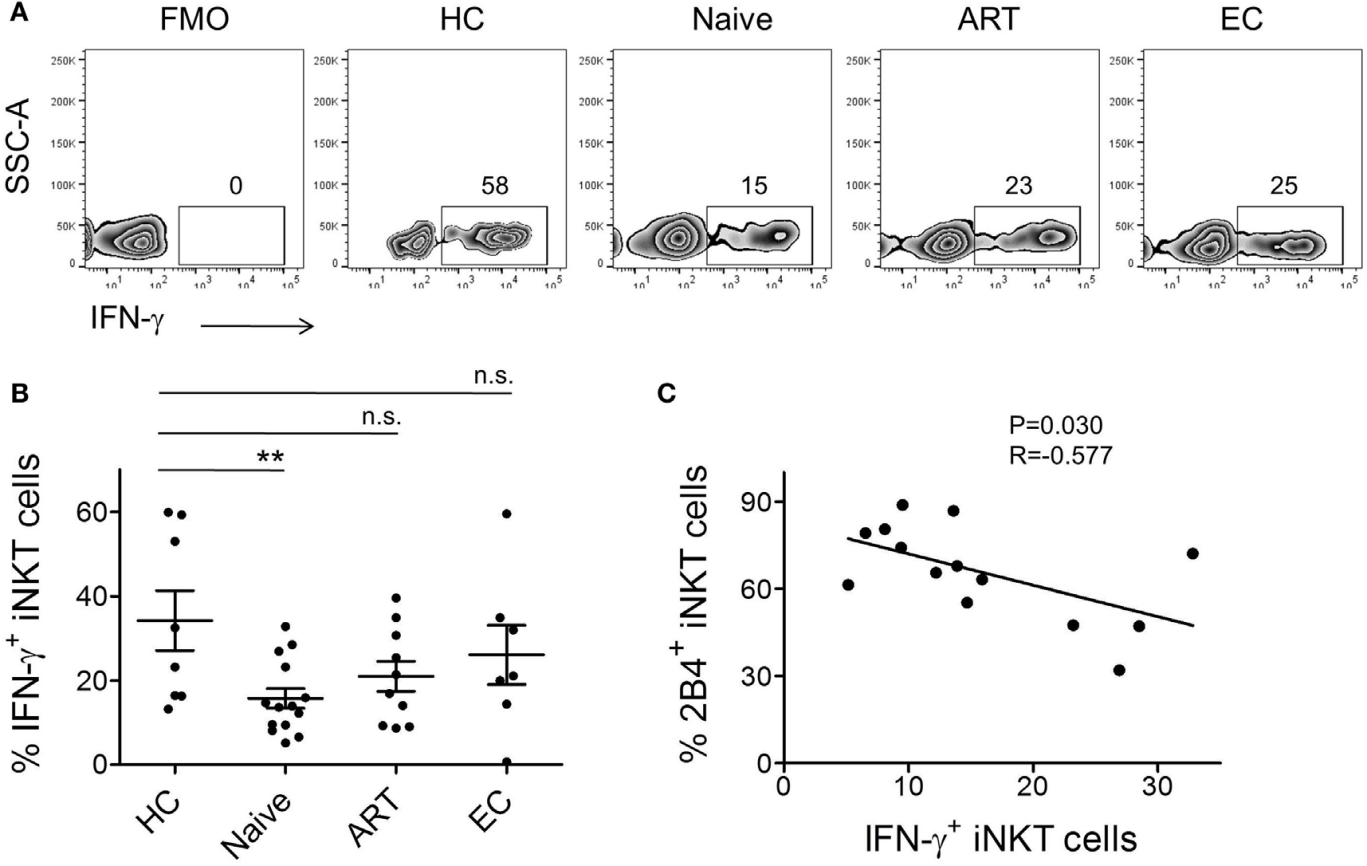
**Invariant natural killer T (iNKT) cell production of IFN-γ and their association with 2B4 levels**. **(A)** Representative plot from each cohort showing percentage of intracellular IFN-γ expression. α-GalCer-stimulated peripheral blood mononuclear cells (PBMCs) were surface-labeled with PBS-57 loaded/CD1d tetramer and CD3 followed by intracellular staining with anti-IFN-γ antibody as described in the section “Materials and Methods.” iNKT cell-gated population were looked for IFN-γ production. **(B)** Scatter plots show mean percentages of IFN-γ production by each cohort HCs (*n* = 8), ART naïve (*n* = 14), ART (*n* = 10), and ECs (*n* = 6). **P < 0.01 and n.s. stands for non-significant. **(C)** Plot showing the correlation between 2B4^+^ iNKT cells and IFN-γ production by ART-naive cohort (*n* = 14).

Rapid production of large amounts of IFN-γ is the hallmark of activated iNKT cells. Hence, we assessed the *ex vivo* ability of peripheral iNKT cells to produce IFN-γ post α-GalCer stimulation. We performed intracellular IFN-γ cytokine staining of PBMCs obtained from the various study groups. After surface staining, cells were permeabilized and subsequently labeled with anti-IFN-γ antibody. Cells were gated on CD3^+^ CD1d-tetramer^+^ population and investigated for IFN-γ production (Figure 4A). As compared to HCs (mean 34.23 ± 7.12%), we observed ~2-fold lower production of IFN-γ by iNKT cells of ART-naïve individuals (*n* = 15, mean 15.74 ± 2.33%, *P* = 0.0069) (Figure 4B). ART-treated individuals, despite the on-going residual immune reconstitution, failed to produce optimal levels of intracellular IFN-γ.

Next, to understand the impact of increased levels of 2B4 on intracellular IFN-γ production, we surface-stained the cells for 2B4 and performed standard 6-h stimulation experiments. However, we were unable to detect 2B4 surface expression during cell acquisition, which most likely could be due to the downregulation of 2B4 after α-GalCer stimulation as shown previously in the case of CD8^+^ T cells, where a similar observation ensued following antigenic stimulation (37). Therefore, in an attempt to understand the potential relationship between 2B4^+^ and IFN-γ^+^ iNKT cells, we performed a correlation analysis. Interestingly, we observed a significant inverse correlation (*r* = −0.577, *P* = 0.030, Figure 4C). These data indirectly indicate the association between the levels of 2B4 and iNKT cell functionality.

### Higher Expression of 2B4 on iNKT Cells Correlated with HIV Disease Progression

**Figure 5:**
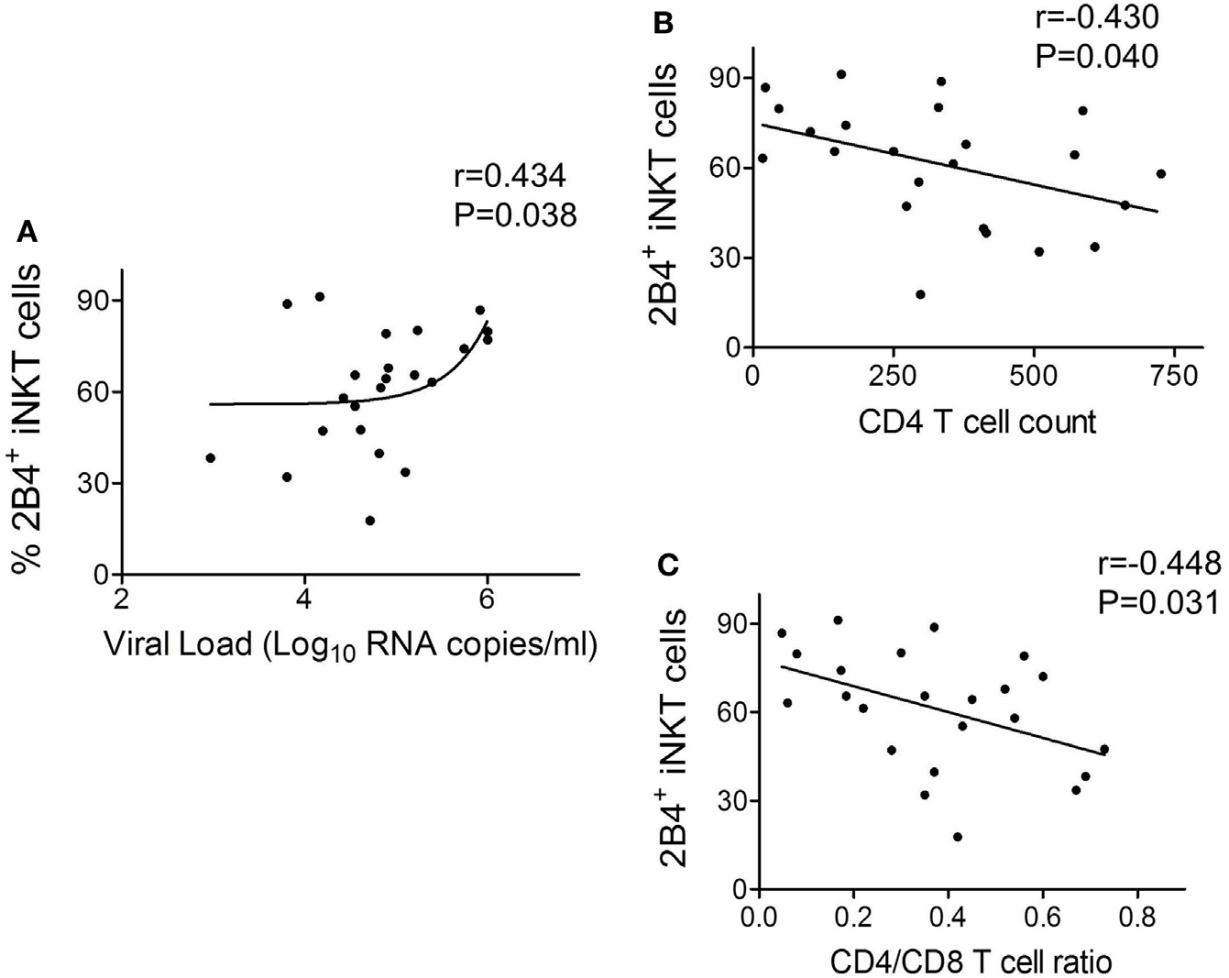
**Correlation between frequency of 2B4^+^ iNKT cells and human immunodeficiency virus (HIV) disease progression**. **(A)** Correlation plot between frequency of 2B4^+^ iNKT cells versus HIV viral load (*n* = 23) **(B)** CD4 counts (*n* = 23) and **(C)** CD4/CD8 ratio (*n* = 23).

In order to understand the relevance of 2B4 expression on iNKT cells with HIV disease, a correlation analysis was performed between 2B4^+^ iNKT cells of ART-naïve cohort and known clinical parameters of HIV disease progression such as viral load, CD4 count, and CD4/CD8 ratio. We found a positive correlation (*r* = 0.434, *P* = 0.038) between 2B4^+^ iNKT cell frequency and the viral load (Figure 5A). Similar analyses were performed with CD4 counts and CD4/CD8 ratio. Notably, we observed an inverse correlation (*r* = −0.430, *P* = 0.040) between 2B4^+^ iNKT cells and CD4 counts (Figure 5B), and CD4/CD8 ratio (*r* = −448, *P* = 0.031) (Figure 5C). Taken together, our data suggest that high levels of 2B4 expression could likely determine the functionality of iNKT cells in HIV infection.

## Discussion

Negative checkpoint regulators, also known as negative regulatory molecules, play a key role in shaping T-cell immune responses against chronic viral infections. A vast majority of previous studies are based on molecules expressed by virus-specific CD8^+^ T cells both *in vivo* (36) and *in vitro* (34, 35, 38). However, a similar observation is lacking for innate lymphocytes, such as iNKT cells. Here, we report for the first time, the relationship between the levels of 2B4, a co-inhibitory molecule, and their impact on iNKT cell dysfunction in HIV infection. Using a large cohort of HIV-seropositive ART-naïve, ART-treated, and ECs, we examined the phenotypic and functional alterations across the peripheral iNKT cell compartment. Here, we observed an upregulation of 2B4 on CD1d-restricted iNKT cells of ART-naïve individuals. Among the iNKT cell subsets, CD4^+^ expressed significantly higher 2B4 levels as compared to the CD4^−^ phenotypes. We also found the existence of a strong association between 2B4 expression and loss of CD4^+^ iNKT cells. Further, the 2B4^+^ iNKT cells of ART-naïve cohort positively correlated with HIV viral load and inversely with CD4 count and CD4/CD8 ratio. Finally, we also found that the iNKT cell phenotypes were functionally impaired in their ability to produce the intracellular anti-viral cytokine IFN-γ whose levels inversely correlated with the expression of 2B4.

Recent studies have shed light on the anti-viral functions of iNKT cells in HBV, HCV, and HIV infections (2). With regard to HIV, there appears to be a rapid depletion of iNKT cells from the periphery of infected individuals (23). Further, a recent study has shown the early loss of peripheral CD4^+^ iNKT cells post-HIV infection, and reported a more profound depletion than the classical CD4^+^ T cells (33). In addition to overall iNKT cell depletion from the periphery, our study has clearly shown the selective depletion of CD4^+^ iNKT cells as compared to the CD4^−^ subset. It should be noted that CD4^+^ iNKT cell depletion was associated with increased CD4^−^ cell numbers both in HIV-naïve and ART-treated individuals, an interesting observation, which has seldom been reported in the past (22, 39). Since we did not observe similar effects with respect to ECs, our data suggest that in addition to cell death due to direct infection of CD4^+^ iNKT cells (40), on-going viral replication and subsequent immune activation (41) could likely play a role in activation-induced cell death (AICD) of activated iNKT cells. One of the limitations of our study is the scarcity of EC samples, which represents a rare population of HIV-infected individuals. Perhaps, the inclusion of more samples from this cohort could have been more informative. Nevertheless, data obtained from our EC study seemingly reflect iNKT cell behavior during HIV infection. We would like to mention that our cohorts comprised different age range and male/female participants that might impact the iNKT cell frequencies. Nonetheless, we succeeded in keeping the age and sex of HC (median age = 39), ART naïve (median age = 38) very much comparable except ART and EC study groups where median age was slightly higher. Since HC and ART naïve represent the two key study groups to describe the effects of HIV on iNKT cell impairments, and hence, a slight age difference with ART and EC may not greatly affect the outcome of our findings. However, a study involving large cohorts of male and female participants indicates that gender and age do not have an impact on iNKT cell frequencies (3).

Initial studies have suggested 2B4 as a co-stimulatory receptor to enhance NK and CD8^+^ T cell functions (18, 42). However, a number of recent studies have shown 2B4 to act as both activating as well as inhibitory receptor on NK cells (43) and HCV-specific CD8^+^ T cells (20). High-level expression of 2B4 and other inhibitory receptors has also been observed on exhausted LCMV-specific CD8^+^ T cells (44) and HIV (45). Interestingly, blockade of 2B4–CD48 interactions has been shown to restore LCMV (46) and human HBV-specific (47) CD8^+^ T cell effector functions *in vitro* culture. Furthermore, absence of mouse 2B4 has been described to promote NK cell-mediated killing of activated CD8^+^ T cells (48). These data support our finding that high levels of 2B4 expression contribute to iNKT cells inhibition. Generally, the dual function of 2B4 depends on surface expression level and downstream signaling involving SAP. Although the dual role of 2B4-mediated regulation of virus-specific CD8^+^ T cells has recently been reported (20), we are not certain if a similar phenomenon also occurs for CD4^−^iNKT (CD8^+^ and DN) cells, as this subset too exhibits elevated 2B4 expression. Hence, more data are required to address this feature in HIV infection. Nevertheless, the current experimental results point to an inhibitory role for 2B4 expressed on iNKT cells. The strong association between iNKT cell depletion and 2B4 levels, and inverse correlation with IFN-γ production clearly supports this hypothesis. Although we did not show the evidence in support, there is a high likelihood that the CD4^+^ subset could potentially have been infected with HIV (40) exhibiting high levels of 2B4, and that were selectively depleted during HIV infection. However, these studies require further investigation to completely explore the underlying mechanisms.

Our data further showed an impaired ability of ART-naïve iNKT cells to produce IFN-γ. ART-treated individuals failed to restore iNKT cell functions despite years of treatment. We were unable to show the expression of IFN-γ^+^ iNKT cells in standard 6-h *in vitro* stimulation experiments potentially due to down modulation of 2B4 expression post-α-GalCer stimulation. One such study has reported this observation where TCR and 2B4 signals were shown to downmodulate 2B4 expression on T cells of HIV-infected individuals (37). Nonetheless, the correlation plot drawn between 2B4^+^ iNKT cells and IFN-γ^+^ iNKT cells clearly indicates an inverse association, supporting the notion that 2B4 signaling indeed could lead to suppressed iNKT cell functions. This correlation study is based on data obtained from two separate experiments examining 2B4 expression and intracellular IFN-γ production. We were able to include only 14 out of 23 ART-naïve samples representing adequate number of cells to perform both 2B4 and IFN-γ expression. However, due to fewer number of cells in the remaining nine ART-naïve samples, we could only perform 2B4 but not IFN-γ expression.

Of note, the clinical significance and relevance of elevated iNKT cell 2B4 expression are clearly supported by the correlation data. The positive correlation with HIV viral load and negative association with CD4 count and CD4/CD8 ratio further supports our finding that 2B4 indeed could serve as a potential determinant of iNKT cell dysfunction. We speculate that on-going HIV replication in ART-naïve individuals may be one of the factors that drive the enhanced 2B4 expression on iNKT cells hence the functionality, thus warrants further investigation. Further studies are required to understand the mechanisms and signaling events that might trigger iNKT cell inhibition. Potential blockade of 2B4 could provide a clear impetus to understand the factors underlying the restoration of iNKT cell effector functions. Overall, our data have clearly revealed the association of 2B4 in iNKT cell impairment during HIV infection.

## Author Contributions

AA, AK, and EMS conceived and designed the experiments, FA, ES, and AA carried out experiments and data analysis, and documented the findings; YY, HT, and GA selected patient samples and collected the clinical data for patients. AA, FA, EMS, and RS wrote the manuscript; RJ contributed reagents and analysis tools; AK and ML provided critical inputs to the manuscript.

## Conflict of Interest Statement

The authors declare that the research was conducted in the absence of any commercial or financial relationships that could be construed as a potential conflict of interest.

## Supplementary Material

Figure S1 **Gating strategy to identify invariant natural killer T (iNKT) cells and their subsets**. Frozen peripheral blood mononuclear cells (PBMCs) were labeled with PBS-57 loaded/CD1d tetramer, CD3, CD4, and live–dead stain **(A)**. Initially, cells were gated on lymphocytes based on SSC-A and FSC-A followed by exclusion of doublet cells using FS-A and FSC-H **(B)**. Singlet cells were gated for live cells by excluding the live–dead cell stain positive population **(C)**. Live cells were gated on CD3^+^ T cells population **(D)**. CD3^+^ T cells were further gated to identify iNKT cells (CD1d tet^+^ CD3^+^ cells) **(E)**. iNKT cells were further gated to detect CD4^+^ and CD4^−^ subsets **(F)**.

Figure S2 **(A)** Basal levels of 2B4 expression. Part of the data is adapted from Figure 2, 2B4 expression was re analyzed only in healthy donors. CD3^+^ T cell-gated cells were further gated for 2B4 expression on CD4^+^ and CD8^+^ T cell populations. **(B)** Healthy donor data were adapted from Figure 3. CD1d tet^+^ CD3^+^ iNKT cells were gated for 2B4 expression on CD4^+^ and CD4^−^ subsets.
