# Supplementary material for: Negative Checkpoint Regulatory Molecule 2B4 (CD244) Upregulation Is Associated with Invariant Natural Killer T Cell Alterations and Human Immunodeficiency Virus Disease Progression
Source: Front Immunol. 2017 Mar 27;8:338. doi: 10.3389/fimmu.2017.00338 (PMC5366318; doi:10.3389/fimmu.2017.00338)
Supplement: Figure S1 — Gating strategy to identify invariant natural killer T (iNKT) cells and their subsets. Frozen peripheral blood mononuclear cells (PBMCs) were labeled with PBS-57 loaded/CD1d tetramer, CD3, CD4, and live–dead stain (A). Initially, cells were gated on lymphocytes based on SSC-A and FSC-A followed by exclusion of doublet cells using FS-A and FSC-H (B). Singlet cells were gated for live cells by excluding the live–dead cell stain positive population (C). Live cells were gated on CD3+ T cells population (D). CD3+ T cells were further gated to identify iNKT cells (CD1d tet+ CD3+ cells) (E). iNKT cells were further gated to detect CD4+ and CD4− subsets (F). [file Presentation_1.PDF]

Suppl. Figure S1. Gating strategy to identify iNKT cells and their subsets

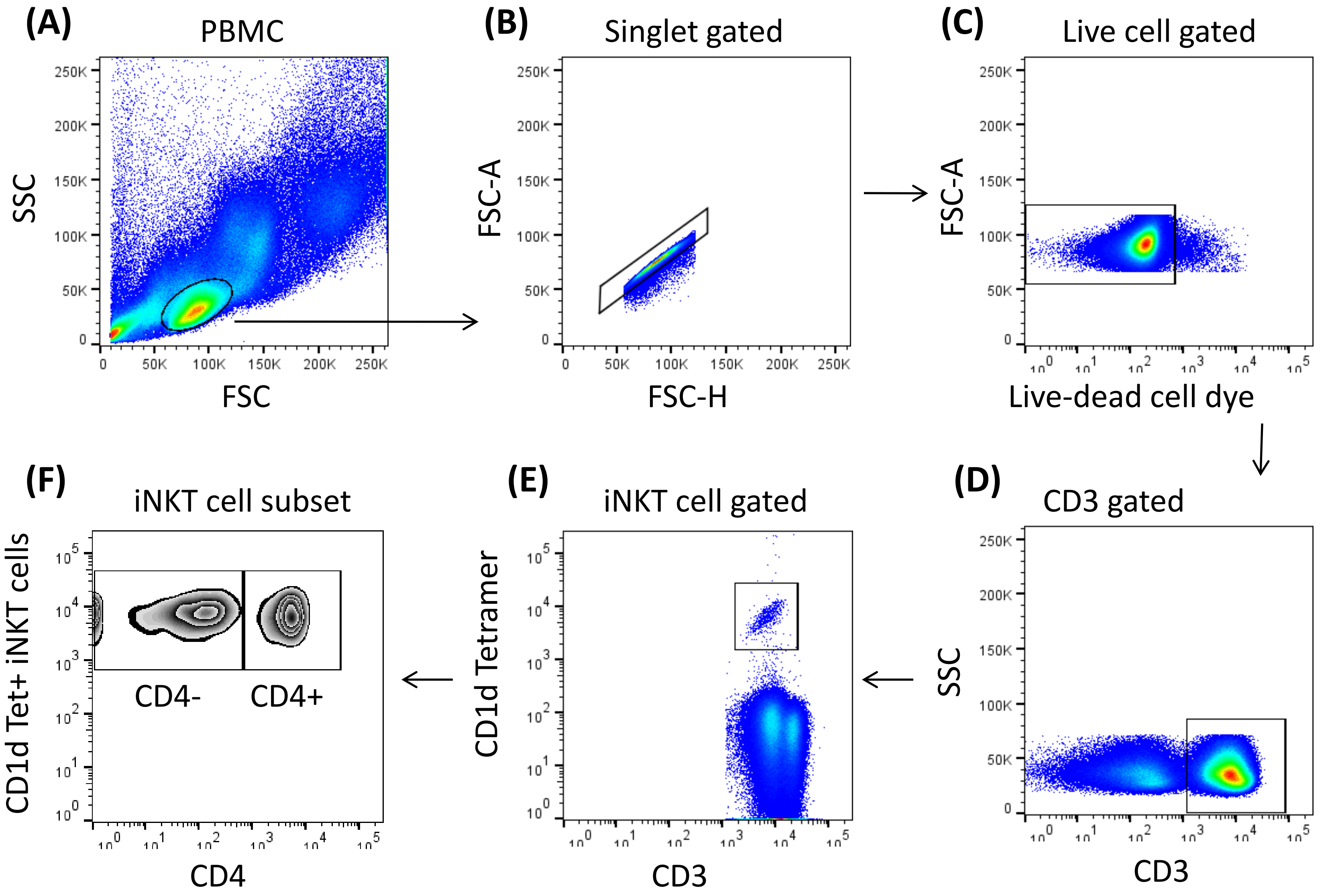

Suppl. Figure S2. Basal levels of 2B4 expression.

(A}

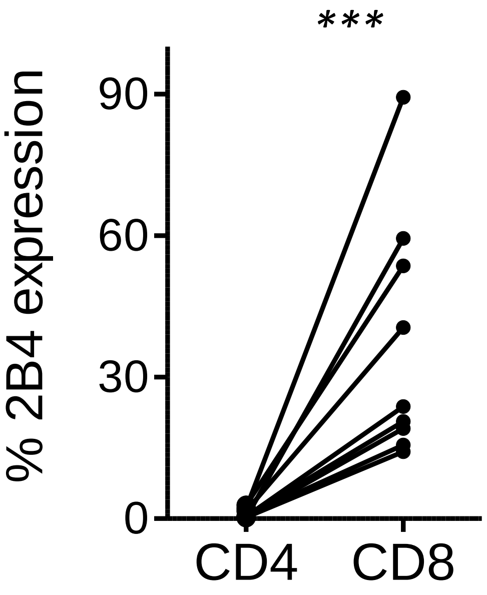

(CD3+ T cell gated)

(B)

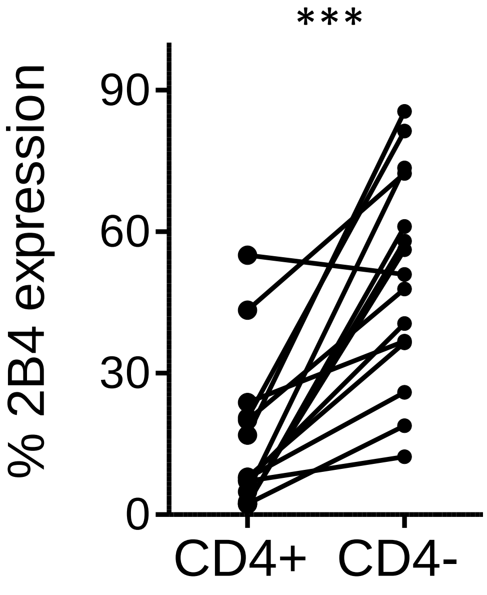

(iNKT cell gated)
